# A Comprehensive Review of Taxane Treatment in Breast Cancer: Clinical Perspectives and Toxicity Profiles

**DOI:** 10.7759/cureus.59266

**Published:** 2024-04-29

**Authors:** Ashish Jivani, Raju K Shinde

**Affiliations:** 1 General Surgery, Jawaharlal Nehru Medical College, Datta Meghe Institute of Higher Education and Research, Wardha, IND

**Keywords:** treatment optimization, clinical perspectives, toxicity, chemotherapy, breast cancer, taxanes

## Abstract

Taxanes, such as paclitaxel and docetaxel, have transformed the landscape of breast cancer treatment, playing pivotal roles in chemotherapy protocols for both early-stage and advanced/metastatic diseases. While these agents have demonstrated remarkable efficacy in enhancing patient outcomes, they are also linked to a range of adverse effects that can impact treatment tolerability and quality of life. This comprehensive review offers an in-depth exploration of taxane therapy in breast cancer, with a focus on clinical perspectives and toxicity profiles. We delineate the mechanisms of action of taxanes, their clinical effectiveness across various breast cancer subtypes, and the prevalent adverse effects encountered in clinical practice. Moreover, we deliberate on strategies for mitigating taxane-associated toxicity and optimizing treatment selection and sequencing based on individual patient characteristics and therapeutic objectives. Finally, we underscore areas for future research and advancement, encompassing the development of novel formulations, the identification of predictive biomarkers for treatment response, and the exploration of combination therapies to bolster therapeutic outcomes. By amalgamating existing evidence and clinical insights, this review aims to apprise clinicians and researchers of the current status of taxane treatment in breast cancer and steer endeavors toward further enhancing patient care and outcomes.

## Introduction and background

Taxanes, including paclitaxel and docetaxel, have become cornerstone drugs in the treatment of breast cancer [1]. They belong to microtubule-stabilizing agents and exert their anticancer effects by disrupting microtubule dynamics, leading to cell cycle arrest and apoptosis. Taxanes are commonly used in both early-stage and advanced/metastatic breast cancer settings, either as part of neoadjuvant or adjuvant chemotherapy regimens or in combination with other targeted therapies [2]. While taxanes have demonstrated significant efficacy in breast cancer treatment, their use is associated with a range of toxicities that can impact patient quality of life (QOL) and treatment outcomes. Understanding these toxicities, along with clinical perspectives such as treatment sequencing, patient selection, and combination therapies, is crucial for optimizing the use of taxanes in breast cancer management. Additionally, insight into taxanes' efficacy and safety profiles across different breast cancer subtypes and patient populations is essential for personalized treatment decision-making [3].

This review aims to provide a comprehensive analysis of taxane treatment in breast cancer, focusing on both clinical perspectives and toxicity profiles. By synthesizing existing literature and clinical evidence, we aim to elucidate the efficacy, safety, and challenges associated with taxane-based therapies in breast cancer. This review also seeks to identify future research and clinical practice directions to improve further the outcomes of breast cancer patients receiving taxane treatment.

## Review

Mechanism of action of taxanes

Brief Explanation of Taxanes

Taxanes represent a class of diterpenes initially discovered in plants of the Taxus genus, particularly yews, characterized by a taxadiene core [4]. This group of antineoplastic agents exhibits a distinct mechanism of action by inhibiting mitosis, disrupting microtubule function, and impeding depolymerization, thereby halting the cell division process [5]. Utilized in the treatment of various cancers, including breast, lung, esophageal, prostate, bladder, and head and neck cancers [5], three taxanes are commonly employed in clinical practice: paclitaxel (Taxol), docetaxel (Taxotere), and cabazitaxel (Jevtana) [5]. Despite their efficacy, taxanes pose challenges in synthesis due to their numerous chiral centers, originally derived from natural sources such as the Pacific yew tree for paclitaxel and the European yew for docetaxel [4,5]. Nevertheless, advancements have led to synthesizing some taxanes, and recent studies have even identified taxanes in the shells and leaves of the common hazel plant [4]. Typically administered intravenously every 1 to 3 weeks, taxanes are often synonymous with taxoids [5]. While generally well-tolerated, taxane therapy may lead to serum enzyme elevations and, albeit rarely, clinically apparent liver injury, which can manifest severely and potentially result in acute liver failure and fatality [5].

Interaction With Microtubules

Research studies have underscored the critical role of microtubule interaction in various biological processes. Microtubule dynamics, tightly regulated by stabilizing microtubule-associated proteins (MAPs) like tau and destabilizing factors [6], are pivotal for cellular functions. Taxanes, a class of chemotherapy drugs, exert their action by targeting microtubules. They promote microtubule polymerization, stabilizing and disrupting their natural dynamics [7]. This interference with microtubule dynamics is central to the anticancer effects of taxanes, as it impedes cancer cell division and growth by inhibiting microtubule function [7]. Moreover, investigations into the interaction between microtubules and MAP7 have revealed insights into their atomic-level interaction. Studies have demonstrated an affinity between an extended α-helix and four tubulin monomers, expanding our understanding of how MAPs interact with microtubules [8]. Additionally, research on the interaction between microtubules and actin during the post-fusion phase of exocytosis has elucidated their involvement in various cellular processes, including cell migration, cytokinesis, neurite outgrowth, and phagocytosis [9]. This interaction contributes to the dynamics of cellular processes and can influence the formation of actin stress fibers and focal adhesions, thereby impacting cellular morphology and function [9].

Role in Cancer Cell Death

Taxanes, a class of chemotherapy drugs, are widely employed in the treatment of various cancers, including breast, ovarian, lung, and prostate cancer, owing to their ability to impede cell growth and trigger cell death in cancerous cells. The mechanism underlying taxanes-induced cell death is multifaceted and encompasses several pathways. Among the primary mechanisms is the induction of micronucleation, characterized by the formation of multiple micronuclei within the cell [10]. This phenomenon arises from aberrant mitosis or non-mitotic mechanisms, such as nuclear membrane tearing, which can culminate in irreversible nuclear membrane rupture and subsequent cell demise [10]. Taxanes have been demonstrated to activate numerous caspases, pivotal players in the apoptotic pathway of cell death. Notably, taxane treatment stimulates caspase-3, an executioner caspase, which orchestrates the proteolytic degradation of proteins and cellular structures, culminating in cell death [10]. Additionally, the activation of initiator caspases, including caspase-8 and -9, has been observed in taxane-treated cells, albeit their precise role in cell death induction remains incompletely elucidated [11]. Beyond the apoptotic pathway, taxanes can induce alternative forms of cell death, such as necroptosis and perforin-mediated pore formation and membrane puncture [10]. Necroptosis denotes a programmed form of necrosis elicited by specific signaling pathways. At the same time, perforin-mediated pore formation and membrane puncture entail the creation of pores in the cell membrane, triggering the release of cellular contents and eventual cell demise [10].

Clinical efficacy of taxanes in breast cancer treatment

Taxane-Based Chemotherapy Regimens

Taxane-based chemotherapy regimens represent a cornerstone treatment modality for breast cancer, markedly enhancing progression-free survival and overall survival rates among patients [12]. Nonetheless, their administration is associated with a heightened risk of specific side effects, such as febrile neutropenia manifested by a diminished white cell count accompanied by fever [12]. Integration of taxanes into anthracycline-based regimens for early breast cancer has been demonstrated to augment survival outcomes and diminish the likelihood of cancer recurrence compared to chemotherapy devoid of taxane components [13]. However, taxane-based chemotherapy concurrently elevates the risk of breast cancer-related lymphedema (BCRL), attributed to tissue remodeling and fibrosis, culminating in chronic swelling of the arms, breasts, or torso [12]. Despite ongoing debate, certain studies indicate a negligible impact of taxane-based chemotherapy on BCRL risk, while others contend that it plays a pivotal role in its onset [12]. In the realm of metastatic breast cancer, taxane-based chemotherapy is advocated as a standard regimen by NCCN guidelines, with both paclitaxel and docetaxel showcasing noteworthy efficacy in HER2 mBC [13]. Beyond breast cancer, taxanes find utility in treating ovarian cancer, prostate cancer, and assorted malignancies, exerting their therapeutic effect by impeding cell division and inducing cancer cell death, thereby impeding tumor progression [14]. Among the taxane arsenal, paclitaxel and docetaxel emerge as prominent options, each characterized by distinct safety profiles necessitating varied premedications before administration [13].

Efficacy in Different Breast Cancer Subtypes

The efficacy of taxanes in treating different subtypes of breast cancer exhibits considerable variation. While the efficacy of anthracycline chemotherapy demonstrates no notable discrepancy among breast cancer subtypes, the specific responses to the combination of anthracyclines and taxanes vary significantly across subtypes [15]. For instance, patients with triple-negative breast cancer (TNBC) characterized by the BL1 or MSL subtype tend to exhibit a higher rate of pathological complete response (pCR). In contrast, those with the LAR and BL2 subtypes display lower sensitivity to the combination regimen, with BL2 subtype patients recording a pCR rate of 0% [15]. In advanced TNBC, taxane-based combination therapy has been proven to substantially extend progression-free survival and overall survival compared to taxane monotherapy, underscoring its effectiveness in managing advanced TNBC cases [16]. However, this combination therapy may heighten the risk of vomiting and diarrhea. However, no statistically significant differences were noted in the complete response rate, objective response rate, disease control rate, and progressive disease indexes [16]. Overall, the efficacy of taxanes in breast cancer treatment is markedly influenced by the specific breast cancer subtype, with varying responses observed across different subtypes. Notably, distinct sensitivities to taxane-based regimens are evident within triple-negative breast cancer subtypes, emphasizing the imperative of personalized treatment strategies tailored to the molecular characteristics of the cancer.

Clinical Trials and Evidence Supporting Taxane Use

Clinical trials and accumulating evidence consistently affirm the efficacy of taxanes in breast cancer treatment. Numerous randomized controlled trials have corroborated the effectiveness of taxanes, including paclitaxel and docetaxel, in bolstering survival rates and mitigating the risk of cancer recurrence across early-stage and metastatic breast cancer cohorts [3,17,18]. Specifically, these trials underscore the substantial risk reduction in cancer recurrence and mortality conferred by taxanes when employed as adjuvant chemotherapy, particularly in cases of early or operable breast cancer, notably in high-risk scenarios [18]. Moreover, taxanes exhibit utility across diverse breast cancer subtypes, including HER2-positive and triple-negative cases, underscoring their versatility in breast cancer management [19]. Taxanes demonstrate a well-tolerated safety profile, albeit side effects such as neutropenia, febrile neutropenia, fatigue, diarrhea, stomatitis, and edema may manifest [18]. Given these considerations, the administration of taxanes necessitates meticulous deliberation, especially in older patients grappling with advanced disease, who may necessitate dose adjustments to mitigate side effects and enhance the quality of life [3].

Toxicity profiles of taxanes

Common Adverse Effects

Hematologic toxicity: Taxanes, such as paclitaxel and docetaxel, are widely utilized chemotherapy agents for treating various solid malignancies, including breast, ovarian, non-small cell lung, and head and neck cancers [20]. Their mechanism of action involves the inhibition of microtubule formation during cell division, resulting in cell cycle arrest at the G2/M phase and activation of cellular apoptosis pathways [20]. Despite their efficacy, taxanes are associated with notable toxicities, including hypersensitivity reactions, myelosuppression, and neuropathy, which often restrict their usage [21]. Hematologic toxicity, particularly neutropenia, is a frequent adverse effect of taxanes, with the incidence varying between different agents, with paclitaxel generally exhibiting less hematological toxicity than docetaxel [22]. Neutropenia can progress to febrile neutropenia, a severe complication necessitating hospitalization and antibiotic therapy [20]. The risk of febrile neutropenia is higher with docetaxel than paclitaxel, often prompting the prophylactic administration of antibiotics to mitigate this risk [20]. Additionally, taxanes may induce anemia, thrombocytopenia, and leukopenia, heightening the risk of bleeding, infection, and fatigue [20]. Management strategies for these toxicities typically involve supportive care measures such as blood transfusions, growth factors, and antibiotics [20].

Neurotoxicity: Taxane-induced neurotoxicity is a prominent and clinically relevant side effect observed in patients undergoing taxane treatment, affecting a substantial proportion of individuals, particularly those treated with paclitaxel [23]. This neurotoxicity manifests predominantly as painful peripheral neuropathy, the most dose-limiting side effect of taxanes, and emerging central neurotoxicity characterized by cognitive impairment and encephalopathy [23]. A significant percentage of patients, ranging from 60% to 90%, develop mild to moderate neuropathy, with up to 30% experiencing severe neuropathy [24]. Symptoms include sensory disturbances such as burning dysesthesia, numbness, tingling, and shooting pains, often distributed in a stocking-glove pattern [25]. While motor neuropathy is less common and typically mild, it seldom interferes with daily activities [25]. The impact of neurotoxicity on QOL still needs to be completed, necessitating comprehensive data collection using validated instruments [25]. Effective management of taxane-induced neurotoxicity often entails dose adjustments guided by therapeutic drug monitoring (TDM), which has been shown to enhance treatment outcomes and minimize toxicity [26].

**Figure 1:**
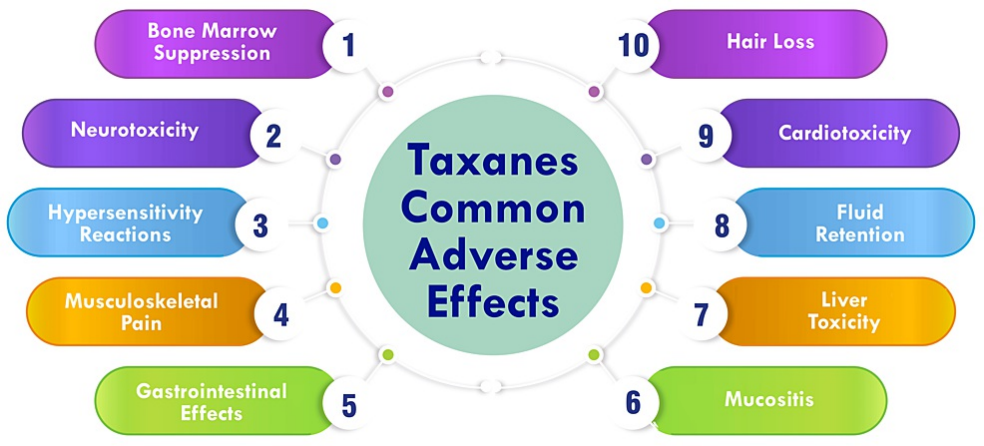
Taxanes' common adverse effects The image is created by the corresponding author.

Gastrointestinal toxicity: Gastrointestinal toxicity is a prevalent adverse effect of taxane-based chemotherapy, with variations observed among different patient populations [27]. Tunisian patients, for example, exhibit higher frequencies of digestive and nail toxicities and lower frequencies of hematological toxicities compared to other populations [27]. In cases where multiple drugs demonstrate similar efficacy in advanced malignant disease, toxicity and QoL considerations become increasingly significant [28]. While both paclitaxel and docetaxel have comparable efficacy, differences in toxicity profiles have been noted between regimens employing these two agents [28]. Gastrointestinal toxicity, encompassing symptoms such as nausea, vomiting, diarrhea, and constipation, is a common manifestation of taxane-based chemotherapy [28]. Although microscopic findings associated with paclitaxel chemotherapy toxicity have been documented, their specificity remains unclear [29]. Taxane therapy is further related to side effects, including peripheral neuropathy, myelosuppression, arthralgias, myalgias, and skin reactions, all of which can impact patients' quality of life [30]. Figure 1 shows taxanes' common adverse effects.

Management of Taxane-Related Toxicity

Prophylactic measures: To mitigate taxane-related toxicity, prophylactic measures are crucial components of management strategies. Peripheral neurotoxicity (NTX) and myelosuppression are two significant types of toxicity associated with taxanes [31]. For peripheral neurotoxicity, proactive measures such as neurological monitoring have shown promise in reducing the incidence of bortezomib-induced peripheral neuropathy in multiple myeloma patients [20]. Cryotherapy and nail solution (NS) have also effectively prevented taxane-induced nail toxicity [32]. In addressing myelosuppression, granulocyte colony-stimulating factors (G-CSFs) can stimulate white blood cell production and mitigate neutropenia [33]. Furthermore, ongoing efforts to develop new analogues and formulations with improved therapeutic and toxicity profiles, including enhanced solubility, aim to enhance the safety and efficacy of taxane-based therapies [31].

Supportive care strategies: Taxanes, while effective in cancer treatment, are accompanied by a spectrum of toxicities, ranging from neutropenia to gastrointestinal issues [31,34]. A multidisciplinary approach involving oncologists, nurses, and pharmacists is paramount to managing taxane-related toxicities effectively. Initial steps involve identifying and assessing the severity of toxicity, followed by appropriate interventions tailored to individual needs [20]. Management strategies encompass using G-CSFs for neutropenia, medications such as gabapentin and pregabalin for neuropathy-associated pain, and physical therapy for maintaining muscle strength and range of motion [20]. Hypersensitivity reactions may be managed with antihistamines, corticosteroids, and H2 blockers, while alopecia may necessitate supportive measures such as wigs or head coverings [20]. Dietary modifications and anti-nausea medications are commonly employed for gastrointestinal issues [20]. Additionally, the advent of orally available formulations with improved pharmacokinetic profiles offers a promising avenue to enhance convenience and reduce traditionally associated toxicities [34].

Dose modification and treatment discontinuation: Dose modification and treatment discontinuation strategies are pivotal in managing taxane-related toxicities, contingent upon the severity of adverse events and the patient's overall health status. Dermatological adverse events like alopecia may not necessitate specific treatment or dose adjustments [35]. However, supportive measures like scalp cooling or topical minoxidil applications may ameliorate hair loss-related distress [35]. For neuropathy, severity, onset time, and duration of symptoms guide decisions regarding dose modifications or treatment discontinuation [1]. Dose-related neuropathy was observed in studies like CALGB Protocol 9342, indicating the need for dose adjustments in severe cases [1]. Similarly, hypersensitivity reactions may require premedication or dose modifications based on severity [36]. Individualized approaches tailored to patient needs and toxicity profiles are imperative for optimizing taxane-based chemotherapy outcomes.

Clinical perspectives on taxane treatment

Patient Selection and Individualized Therapy

Optimizing the benefits and mitigating the risks associated with taxane treatment in breast cancer necessitates meticulous patient selection and individualized therapy approaches. A patient-level meta-analysis encompassing 100,000 women across 86 randomized trials revealed that for women with low-risk tumors who underwent optimal surgery or radiotherapy and received endocrine or anti-HER2 therapy, the benefits of an anthracycline plus taxane chemotherapy might not outweigh the risks when compared to less toxic regimens [37]. Additionally, an investigation into the association between taxane type and chemotherapy-induced peripheral neuropathy (CIPN) highlighted that factors such as the specific taxane used, dosage, treatment duration, various patient characteristics, age, ethnicity, body mass index, diabetes status, prior history of neuropathy, treatment stage, and chemotherapy regimen-significantly influence the incidence and severity of CIPN [38]. Furthermore, a comprehensive literature review focusing on taxanes in breast cancer treatment underscores the importance of comprehending their bioactivity, optimal delivery methods, and synergistic potential when combined with other therapeutic agents to maximize their clinical efficacy [39].

Combination Therapies With Taxanes

The investigation of combination therapies involving taxanes has been extensive across various cancer types, encompassing breast, prostate, and esophageal cancers. Particularly in metastatic breast cancer, combinations of bevacizumab and taxanes have exhibited superior efficacy compared to single-agent taxanes in the initial treatment, leading to enhanced survival outcomes and diminished cancer progression risk across diverse patient subgroups [40]. In prostate cancer, the exploration of taxane-based combination regimens has also been notable. Docetaxel has been coupled with additional agents such as prednisone and, more recently, innovative hormonal therapies like abiraterone, enzalutamide, and apalutamide, improved survival rates and enhanced disease control [41]. However, it is crucial to acknowledge that while taxane-based combination therapies offer therapeutic benefits, careful consideration must be given to the associated elevated risk of side effects and toxicities. Notably, taxanes may induce febrile neutropenia, a potentially life-threatening complication, necessitating supportive interventions to manage these adverse effects effectively [42].

Impact on Treatment Sequencing and Duration

The sequencing and duration of taxane administration are pivotal considerations across various cancer treatments, with a profound impact on treatment outcomes. Both research endeavors and clinical perspectives underscore the necessity of optimizing the sequence in which taxanes are integrated with other chemotherapy agents to attain optimal therapeutic results. Investigations spanning different cancer types, including gastric and breast cancer, underscore the imperative for tailored approaches that align with individual patient characteristics and treatment objectives [43-45]. In neoadjuvant chemotherapy for breast cancer, the sequence order of anthracyclines and taxanes garners significant attention, with studies aiming to elucidate the optimal sequencing to enhance treatment efficacy while mitigating the risk of disease recurrence and mortality [44,45]. Moreover, the duration of taxane-based therapy, particularly in metastatic breast cancer, assumes paramount importance in striking a balance between treatment benefits and potential side effects, such as taxane-induced peripheral neuropathy (TIPN). The onset of TIPN can profoundly impact patients' quality of life and their ability to engage in daily activities [46]. Furthermore, selecting taxane-based regimens across different treatment lines, whether first-line or second-line therapy, necessitates meticulous consideration of factors, including patient compliance, treatment-related toxicity, and the availability of alternative therapeutic options to ensure optimal treatment outcomes [43]. The exploration of taxane sequencing in conjunction with other agents, such as anti-angiogenic therapies, is underway to delineate the most efficacious treatment strategies in advanced cancers like gastric cancer [43].

Future directions and challenges

Emerging Research on Taxane-Based Therapies

**Figure 2:**
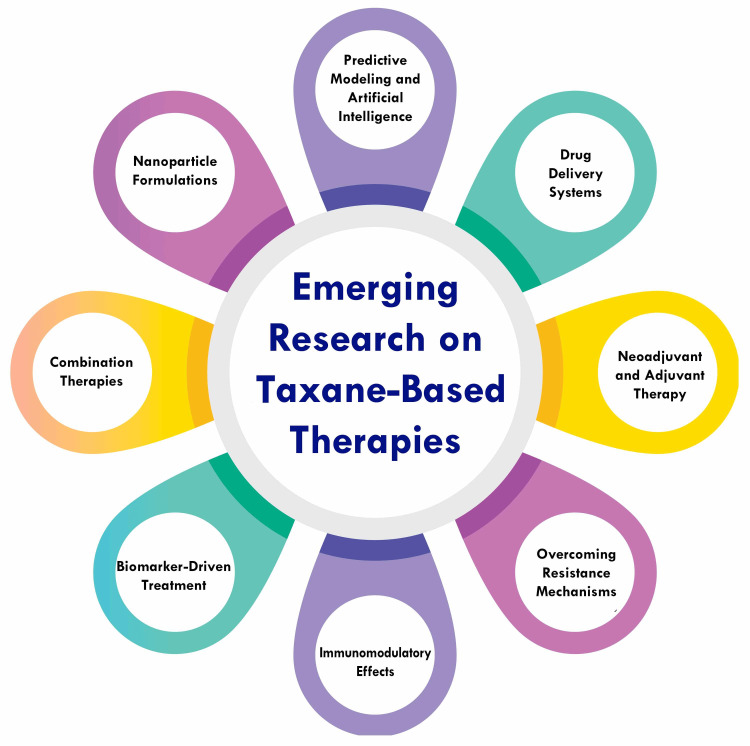
Emerging research on taxane-based therapies The image is created by the corresponding author.

Emerging research on taxane-based therapies for breast cancer is directed toward optimizing their utilization, surmounting resistance, and enhancing their safety and efficacy. One avenue of exploration involves the development of nanotechnology-based combination therapies aimed at augmenting taxane delivery and circumventing drug resistance [47]. Additionally, investigators are delving into the synergy between taxanes and other agents, such as trastuzumab, to bolster therapeutic outcomes and enhance patient prognosis [48]. Another focal point is the evaluation of novel taxanes and their derivatives, which hold promise for heightened efficacy and diminished side effects compared to current formulations. Cabazitaxel, a recent addition to the taxane family, has exhibited encouraging outcomes in the treatment of metastatic breast cancer, particularly among patients who have developed resistance to other taxanes [47]. Moreover, there is a burgeoning interest in deciphering the mechanisms underpinning taxane resistance and devising strategies to surmount it. One approach involves pinpointing and targeting specific genetic mutations associated with taxane resistance, such as the overexpression of the P-glycoprotein efflux pump [47]. Furthermore, researchers are exploring the potential of employing taxanes within personalized medicine paradigms, wherein individual genetic profiles and tumor characteristics guide treatment selection. Such tailored approaches promise to optimize therapy for individual patients, thereby enhancing treatment outcomes [45]. Figure 2 shows emerging research on taxane-based therapies.

Addressing Resistance Mechanisms

Addressing resistance mechanisms in breast cancer treatment involving taxanes stands as a critical domain of investigation. Taxanes, a class of chemotherapy drugs with a history of over 40 years in treating both early-stage and advanced/metastatic breast cancer, exert their therapeutic effect by impeding cancer cell division and growth, chiefly through interference with microtubules integral to the cell's structural integrity and replication machinery [19]. Nevertheless, not all breast cancers respond favorably to taxanes, and resistance to these drugs can emerge, notably in triple-negative breast cancer [19]. Researchers are diligently striving to unravel the underlying causes of this phenomenon and devise strategies to circumvent it. One avenue of exploration entails delving into combination therapies to augment taxane efficacy and surmount resistance. For instance, the synergistic potential of combining taxanes with other agents, such as Erlotinib or Penfluridol, has exhibited promise in addressing resistance mechanisms in breast cancer cells [42]. Furthermore, alternative production methods for taxanes, such as endophytic fungi-based production, are under scrutiny, offering the potential for environmentally friendly and cost-effective solutions [42]. Advancing toward personalized medicine approaches in breast cancer treatment, including taxanes, represents a promising trajectory. Tailoring therapies to individual patients based on their unique genetic makeup and tumor characteristics can potentially optimize treatment efficacy and outcomes [49]. Such strides in research and clinical practice are pivotal in navigating the complexities of taxane resistance and elevating the standard of care for breast cancer patients.

Novel Formulations and Delivery Strategies

Various innovative approaches are being explored to advance breast cancer therapy, with nanotechnology-based strategies at the forefront. Utilizing nanoparticles (NPs), these approaches offer heightened drug loading capacity, reduced toxicity, and enhanced stability compared to conventional chemotherapy agents [50]. NPs hold the potential to precisely target malignant cells, thereby improving drug delivery efficiency and minimizing off-target effects. Receptor-based targeting represents another promising avenue in breast cancer treatment. By directing therapeutic agents towards specific receptors such as estrogen receptors (ERs), progesterone receptors (PRs), and human epidermal growth factor receptor 2 (HER-2), researchers seek to enhance treatment precision and efficacy [50]. This approach holds particular relevance in personalized medicine paradigms.

Inorganic drug delivery approaches, exemplified by gold nanoparticles (GNPs), have garnered attention in breast cancer chemotherapy. GNPs, characterized by their diminutive size and specificity, can infiltrate tumour cells, serving as potent biomarkers for cancer diagnosis and facilitating enhanced drug delivery efficiency [50]. The advent of novel taxane formulations, notably nab-paclitaxel, presents alternative options in breast cancer therapeutics. These formulations strive to address limitations such as poor solubility and side effects, thereby enhancing therapeutic outcomes following the failure of conventional combination chemotherapy [51]. Their development marks a significant stride toward improving patient care and treatment efficacy. Despite these advancements, challenges persist in taxane formulations, including issues with aqueous solubility and adverse effects. Researchers are actively exploring alternative formulations utilizing nanocarrier systems such as NPs, liposomes, micelles, and dendrimers [52]. These systems promise to improve drug solubility, augment cellular uptake, and enable targeted drug delivery, thus presenting viable solutions to overcome existing challenges in breast cancer therapy.

## Conclusions

In conclusion, taxanes represent vital components in the treatment arsenal against breast cancer, exhibiting significant efficacy across various disease stages. However, their use comes with a spectrum of toxicities, necessitating vigilant monitoring and management to ensure patient safety and treatment adherence. A nuanced understanding of patient-specific factors, including tumor subtype, disease stage, and individual tolerability should guide clinical decision-making surrounding taxane therapy. Integrating taxanes into multidisciplinary treatment approaches, alongside targeted therapies or immunotherapy, holds promise for optimizing outcomes in select patient cohorts. Looking ahead, ongoing research efforts should focus on unraveling mechanisms of taxane resistance, refining personalized treatment strategies, and exploring novel formulations or delivery methods to enhance therapeutic efficacy while minimizing adverse effects. By addressing these challenges and embracing innovative approaches, we can further advance the role of taxanes in breast cancer treatment and improve the lives of patients facing this challenging disease.
